# Marital Status, Marital Transitions, and Sleep Quality in Mid to Late Life

**DOI:** 10.1093/geroni/igab046.1570

**Published:** 2021-12-17

**Authors:** Kristin August

**Affiliations:** Rutgers University, Camden, Camden, New Jersey, United States

## Abstract

Sleep is an important behavior in the prevention and management of chronic conditions in later life. Marital status may account for variability in sleep quality, but little is known about this association in the later part of life or how transitions into and out of marriage are related to changes in sleep quality. This study used the resource model and crisis model as frameworks to understand how marital status and marital transitions were related to sleep quality in mid to late life and whether these findings differed by gender. Interview data from 2,872 participants 50-74 years old (M=59.77 years; 63.7% women) from the ORANJ BOWL, a longitudinal panel study in New Jersey, were used. Marital status and sleep quality were examined in two waves approximately 10 years apart. All analyses controlled for health and sociodemographic characteristics. Weighted regressions revealed that individuals in committed romantic relationships and women had worse sleep quality than those in other marital status groups and men (p<.005). Weighted fixed effects regressions revealed that compared to individuals who remained married, individuals who remained divorced or widowed or who became widowed had better sleep quality, whereas those who became divorced had worse sleep quality (ps<.05); individuals who transitioned into marriage had better sleep quality than those who remained divorced or widowed (ps<.03). Findings differed depending on the index of sleep quality examined. Efforts to understand which middle-aged and older adults are most vulnerable to sleep disturbances can inform the design of interventions to promote better sleep quality.

